# Postpartum Pubic Symphysis Diastasis: A Case Report

**DOI:** 10.7759/cureus.57648

**Published:** 2024-04-05

**Authors:** Nicole Vilar, Danielle Donahue, Harshita Nadella, Rahil Malik

**Affiliations:** 1 Dr. Kiran C. Patel College of Osteopathic Medicine, Nova Southeastern University, Davie, USA; 2 Department of Obstetrics and Gynecology, HCA Florida Westside Regional Medical Center, Plantation, USA

**Keywords:** prevent complications, fetal macrosomia, pregnancy risk factors, post-partum period, pubic diastasis

## Abstract

We present the case of a 25-year-old African American female patient (G1P0) with a past medical history of brain arteriovenous malformation repair, pneumonia, and a urinary tract infection who was admitted to the labor and delivery floor at 39 weeks for a spontaneous vaginal delivery of a 4.025 kg female baby. In the immediate postpartum (PP) period, the patient presented with severe pelvic pain and trouble ambulating. Conservative management of oral non-narcotic analgesics was initiated until the diagnosis of PP pubic symphysis diastasis (PSD) was made. Due to the persistence of pelvic pain, the patient underwent a pubic symphysis joint steroid injection and was discharged on day 8. Within 24 hours of discharge, the patient was readmitted to the emergency department with severe pain and an inability to walk. Her pain was managed conservatively with intravenous narcotics and non-steroidal anti-inflammatories, which quickly dissipated the pain. She was observed and discharged once she reported improvement in pain, and she was reassessed five days later at her obstetrician’s clinic. In the clinic, the patient presented with mild tenderness in the pubic symphysis region but demonstrated improvement in her antalgic gait with an ability to walk and urinate without difficulty. Despite a lack of follow-up imaging, the patient was reassured that her PSD and associated tenderness should completely resolve within three to four months.

## Introduction

During pregnancy, the pubic symphysis, a cartilaginous joint, widens to assist in childbirth to a maximum physiological width of approximately 1 cm (10 mm) [1]. During childbirth, the hormone relaxin is responsible for helping one’s ligaments stretch, typically to help the fetus pass through the pelvic canal with ease. A widening exceeding that physiological width during childbirth leading to debilitating pain postpartum (PP) is when the diagnosis of pubic symphysis diastasis (PSD) should be considered. The incidence of complete separation is reported to be within 1 in 300 to 1 in 30,000, with many instances likely undiagnosed [1]. It is important to keep in mind that undertreated PSD can lead to further pelvic issues in the future, such as urinary incontinence, pelvic organ prolapse, and diastasis recti. This further highlights the need to promptly diagnose and treat this condition [1].

The most common way that this can happen is during childbirth; however, there are other etiologies, such as trauma, horseback riding, sports injuries, or a motor vehicle accident. Several risk factors may predispose an individual to this type of injury, such as primigravida, prolonged labor, multiple gestations, forceps delivery, epidural anesthesia, and labor or macrosomic fetus (4,000 gm) [1].

Although several cases have been documented within the PP period, many of those stand to be outside the United States and without any prior medical history. Therefore, we present a case of PSD in a 25-year-old African American female patient (G1P0) with a robust prior medical history during pregnancy that was not definitively diagnosed until the sixth day of her PP period. This case will further discuss the details of the patient’s case, management options, and the role of further imaging for continued surveillance and care of her PSD.

## Case presentation

Patient information and presenting symptoms

A 25-year-old African American female patient (gravida 1, para 0) was admitted to the labor and delivery floor at 39 weeks with laboring pains. Upon admission, the patient had a pregnancy BMI of 34.5 kg/m2 with a pertinent past medical history of a brain arteriovenous malformation repair prior to pregnancy. During pregnancy, the patient’s medical history was also significant for pneumonia and a urinary tract infection, which were treated without further complications prior to labor. However, upon admission, cardiology was consulted to evaluate the patient’s heart rate, which was 120 beats per minute. All of her workup was negative, with the plan to repeat the evaluation in the PP phase. Upon admission, fetal monitoring was implemented, misoprostol and dinoprostone were administered for cervical ripening, and a spinal epidural was performed. After nine hours and 30 minutes of laboring, a 4.025 kg female baby was delivered by spontaneous vaginal delivery. During parturition, the patient suffered a second-degree tear that was repaired with a 2-0 chromic suture. In the immediate PP period, the patient complained of localized severe pelvic pain and trouble ambulating; she was only able to ambulate with assistance. Neurology was consulted, and on examination, all cranial nerves, as well as motor and sensory functions, were intact. Normal tone and 2+ deep tendon reflexes in the right (R) and left (L) bicep, R/L tricep, R/L brachioradialis, R/L patella, and the R/L Achilles were noted. The only documented abnormal neurologic finding was an antalgic gait. Despite conservative treatment with non-narcotic analgesics and a pelvic binder, on PP day 3, the patient continued to have difficulty ambulating with persistent pelvic pain.

Diagnostic Studies

**Figure 1:**
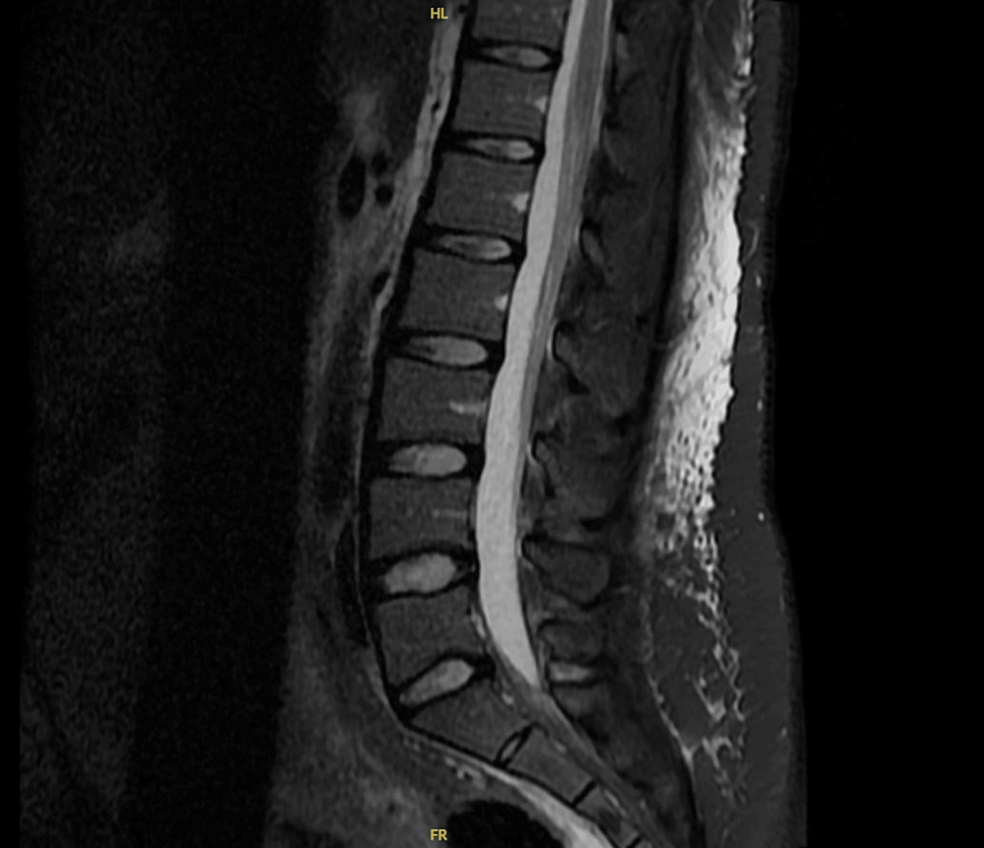
Lumbar MRI This is the lumbar MRI that ruled out lumbosacral origin.

**Figure 2:**
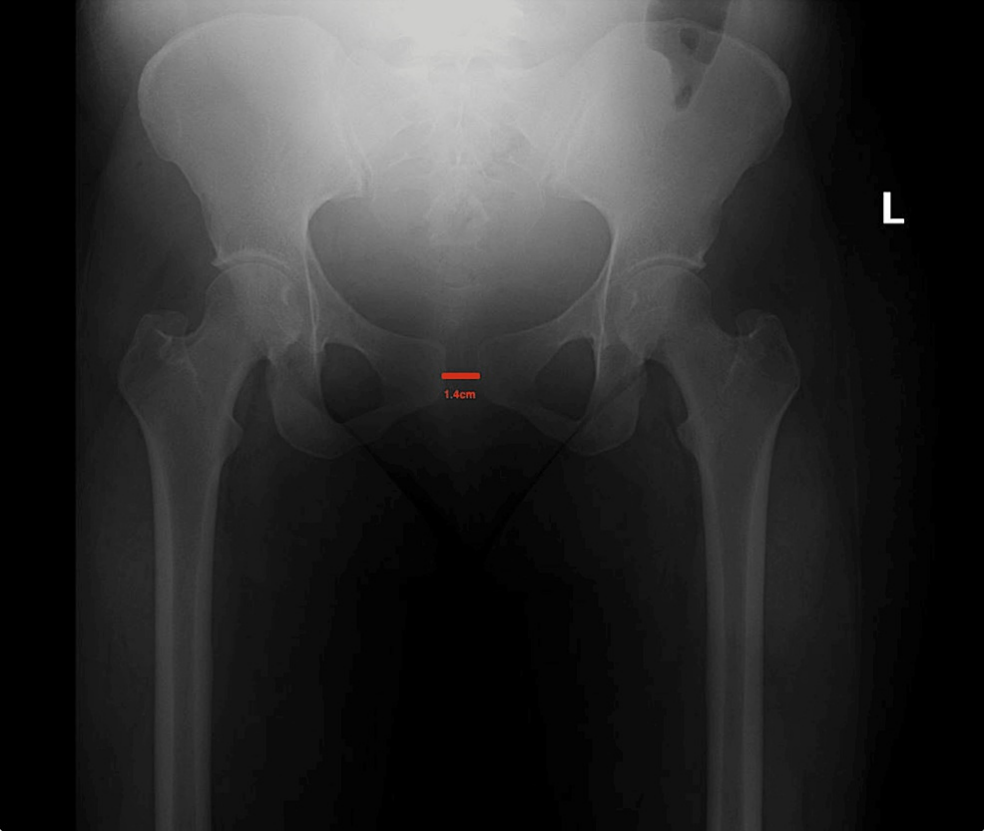
Plain AP pelvic radiograph, demonstrating PSD This is an AP pelvic radiograph of the 1.4-cm pubic diastasis. AP, anteroposterior; PSD, pubic symphysis diastasis

**Figure 3:**
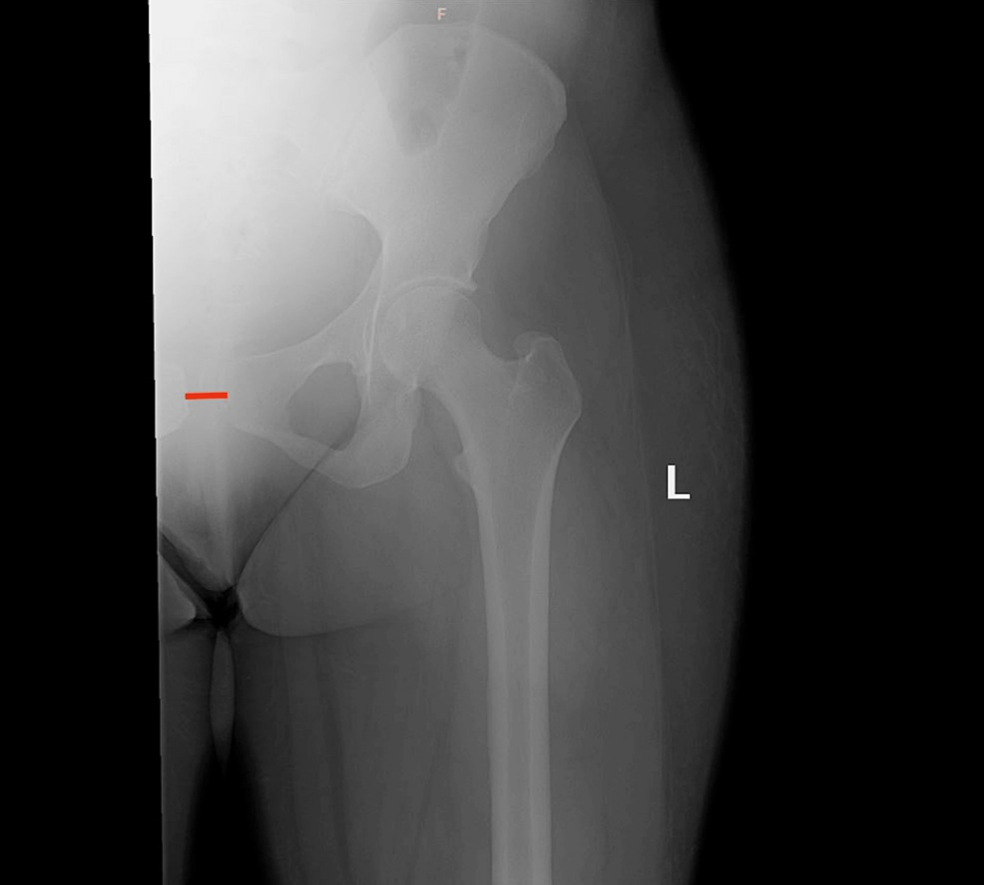
Plain AP radiograph of the left hip This is an AP radiograph of the left hip, demonstrating 1.4-cm pubic diastasis. AP, anteroposterior

To rule out lumbosacral neurologic issues as a possible etiology of her symptoms, a lumbar MRI was performed, and the results obtained were normal (Figure 1). Additionally, an anteroposterior pelvic radiograph (Figures 2, 3) was taken two days later, in which a 14-mm pubic separation was noted. This finding led to the diagnosis of PP PSD on PP day 6.

Treatment

**Figure 4:**
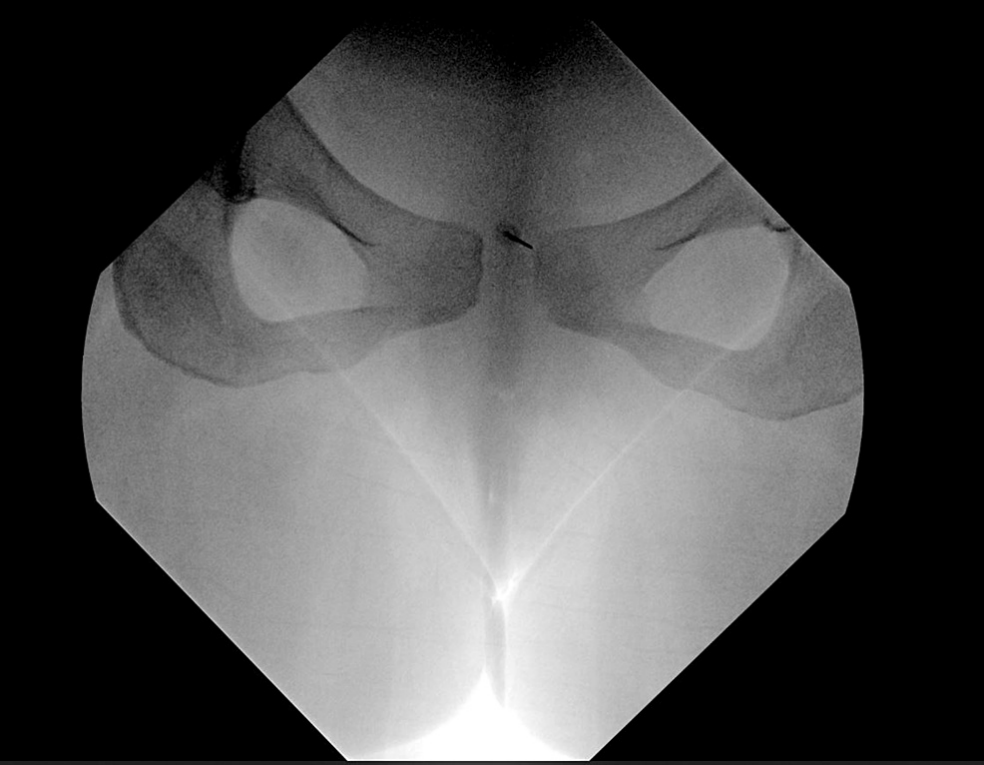
Fluoroscopy-guided lumbar spine MRI with PSI This demonstrates fluoroscopy-guided treatment of the patient’s PSD. PSD, pubic symphysis diastasis; PSI, pubic symphysis injection

The patient was begun on a conservative pain regimen that consisted of oral (PO) non-narcotic analgesics: 15 mg tablet PO of ketorolac tromethamine and 50 mg, 300 mg, and 40 mg capsule PO of butalbital, acetaminophen, and caffeine. However, once the diagnosis was made, a pubic symphysis joint injection with fluoroscopy (Figure 4) was performed the following day, PP day 7. The lumbosacral area was prepped with povidone-iodine three times and draped into a sterile field. The skin overlying the joint was anesthetized with 3 cc of lidocaine 1%. The joint was identified by cephalo-oblique fluoroscopy. The needle position was confirmed through oblique, anteroposterior, and lateral radiographs. Moreover, 2 cc of Omnipaque 200 was injected, confirming intraarticular contrast spread. A combination of 4 cc of Lidocaine 2% and 10 mg of dexamethasone was injected into the joint. Upon discharge, the patient was given a pelvic binder and at-home pain management therapy consisting of Tylenol 650 mg PO, 600 mg ibuprofen PO as needed (PRN), and 5 mg oxymorphone PRN.

Outcome

The patient was sent home on PP day 8 as her pain and ambulation both improved, and she only needed a walker to ambulate independently. Incidentally, shortly after discharge, the patient returned to the emergency department with a pain recurrence and an inability to walk. On examination, there was tenderness to palpation of the pelvic area, which was not significantly different from her previous examination before discharge. She denied heavy vaginal bleeding and blood clots. The patient was given an infusion of intravenous ketorolac tromethamine in the emergency room. She was observed and reported improvement in pain, and she was discharged with instructions to follow up with her obstetrician outpatient and continue her physical therapy. The patient was reassessed five days later at her first outpatient PP visit, in which she stated she was asymptomatic and able to walk, urinate, and defecate. On physical examination, there was mild tenderness to palpation around the pubic symphysis. The patient was subsequently advised that her pubic symphysis pain and diastasis would improve within three to four months with the assistance of physical therapy and pain medications. To date, there have been no further radiographs performed to confirm whether there has been improvement in the pubic diastasis.

## Discussion

Peripartum and PP PSD is an uncommon complication of childbirth and is typically successfully managed nonoperatively with bed rest, oral analgesics, pelvic binders, and/or local steroid injections. When refractory to nonoperative management, operative management options for PSD, including open reduction and internal fixation, anterior plating, or external fixation, can be considered under orthopedic care. While PSD is a clinical diagnosis, imaging such as radiography, ultrasound, and MRI is helpful in corroboration and evaluation of the width of the diastasis. The current literature states that the four highly specific and sensitive clinical tests for PSD evaluation include point tenderness on palpation of the joint, a positive FABER test, a positive straight leg raise test, and a positive Trendelenburg test [2]. In this specific case, only one of these was performed; the joint was palpated and did yield point tenderness.

A thorough review of the literature identified multiple factors, such as a small pelvic outlet, large gestational weight, and previous pelvic trauma, that predispose women to PSD [1]. However, a case-control study aimed to determine the statistical significance of these factors and found that the only statistically significant predisposing factor for PSD was nulliparity [3]. Additionally, this study also found that the incidence of PSD increased with advancing maternal age [3]. Other factors theorized to play a role in PSD, such as maternal prepregnancy BMI, maternal diabetes (pregestational or gestational), fetal weight, duration of labor, and vacuum-assisted delivery, were not shown to be statistically significant risk factors [1].

PP PSD can be difficult to diagnose due to variability in reported PP pain, difficulty distinguishing pelvic pain from other surrounding structures, and delay in symptoms due to spinal anesthesia administered to some patients during delivery. The most common reported features of PSD include pain in the anterior pelvis and/or suprapubic area, pain in the hip joints, and/or radiation of pain down the lower extremities. Physical examination may reveal tenderness to palpation in the anterior pelvis, difficulty or inability to ambulate or bear weight, ambulation with a waddling gait, and extreme pain with attempted manipulation of the pelvic region [2]. While the PP onset of PSD is the most common, the literature reveals that patients may not present with symptoms up to three months after PP [2]. When reviewing the literature, most patients with PSD reported symptoms within the first week of PP. Patients who experience PSD during delivery may report acute anterior pelvic pain, an inability to ambulate following delivery, and “feeling a pop” during delivery [4,5].

When PSD occurs, first-line treatment is conservative management with bed rest, pelvic binder, non-narcotic analgesics such as NSAIDS and acetaminophen, and physical therapy. As a clinical diagnosis, PSD can be treated as low risk with these measures. Additionally, some case reports have identified noninvasive soft tissue methods such as transcutaneous electrical nerve stimulation and soft tissue massage as beneficial in reducing pain [6,7]. While these methods have not been studied in a case-controlled environment, they may assist in recovery and pain reduction. If conservative methods fail and/or the separation of the pubic symphysis is ≥3 cm, orthopedic surgical evaluation and possible intervention are recommended. The recovery time for PSD can be lengthy; however, the literature shows that complete resolution of clinical symptoms and near-complete closure of PSD on follow-up radiographs occur in most cases within three months [1].

**Figure 5:**
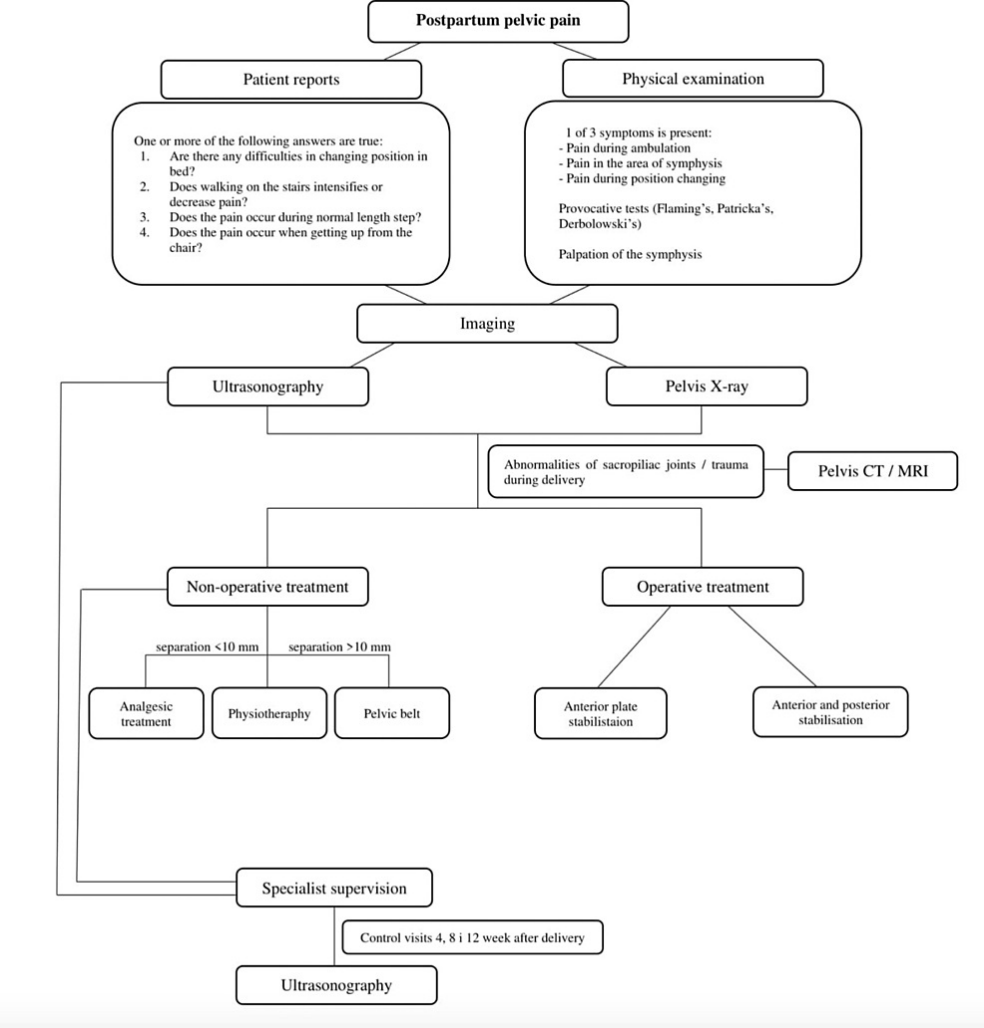
Algorithm for the current consensus of imaging use in evaluation and treatment of suspected pubic symphysis separation Image source: Stolarczyk et al., 2021 [8]; Creative Commons Attribution (CC BY) license

While PSD is a relatively uncommon occurrence, it should remain high on every obstetrician's differential as a potential cause of anterior pelvic pain and difficulty ambulating in PP patients. If PSD is suspected, a pelvic ultrasound scan can be diagnostic and used for screening, but should be followed by an anteroposterior pelvic radiograph [1]. This will allow for the assessment of the degree of separation of the pubic symphysis in a patient presenting with the aforementioned clinical findings (Figure 5). Simultaneously, the sacroiliac joints should be evaluated bilaterally on the plain radiograph for any gross separation [1]. In this patient, it is believed that a delay in diagnosis was likely due to a delay in radiographic imaging and a lack of awareness by the obstetric team in identifying this condition as a possible etiology in the early PP period. Alternate etiologies for pain, including musculoskeletal and neurologic etiologies, were considered. It was not until PP day 3 that a non-contrast MRI of the lumbar spine was ordered. The MRI was unremarkable in identifying etiologies for pelvic pain, and after three days of limited pain improvement despite around-the-clock pain management, a pelvic X-ray was ordered by the incoming physician on-call that identified PSD in this patient. Although the patient was diagnosed within the first week of PP, she could likely have avoided expensive and unnecessary testing if a pelvic radiograph had been ordered within the first three days of the PP period. Awareness and early diagnosis of PSD can lead to early treatment and management that can provide PP patients with early pain relief and recovery for this otherwise uncommon and often underdiagnosed condition. Obstetricians and hospital personnel caring for PP patients should stay current with appropriate guidelines and treatment algorithms for PSD.

## Conclusions

PSD remains a rare, but not impossible, occurrence for patients delivering vaginally. Keeping common risk factors in mind, such as macrosomic fetuses and epidural anesthesia, obstetricians need to maintain a high level of suspicion in women who present with extreme pelvic pain and tenderness during and/or following labor and delivery.

Early recognition, imaging, and management of PSD can accelerate the patient’s recovery process and avoid complications. Furthermore, as discussed above, several adverse effects, such as urinary incontinence and pelvic organ prolapse, can result from poor diagnosis and treatment of PSD. Keeping those in mind, obstetricians should maintain close follow-up during the PP period, as well as maintain communication with orthopedic surgery in cases of suspected PSD. At this time, prevention strategies for PSD have not been identified, which makes it a unique area for future research.
